# Functional Effects of Calcium Sources in Soilless Lettuce Cultivation

**DOI:** 10.3390/plants15142209

**Published:** 2026-07-20

**Authors:** Talys Moratti Lemos de Oliveira, Ana Júlia Câmara Jeveaux Machado, Janyne Soares Braga Pires, Jan da Vitória, Tulio Silva Lara, Lúcio de Oliveira Arantes, Adriano Alves Fernandes, Sara Dousseau-Arantes

**Affiliations:** 1 Federal Institute of Espírito Santo, Barra de São Francisco Campus, Rodovia ES 320, Km. 118, Zona Rural, Barra de São Francisco 29800-000, ES, Brazil; 2 Federal University of Espírito Santo (UFES), Center for Natural Human Sciences (CCHN), Goiabeiras Campus, Vitória/ES. Av. Fernando Ferrari, 514, Vitória 29075-910, ES, Brazil; 3 Institute of Water Science and Technology, Federal University of Western Pará, Tapajós Campus, Rua Vera Paz, Bairro Salé, Santarém 68035-110, PA, Brazil; tulio.lara@yahoo.com.br; 4 Capixaba Institute of Research, Technical Assistance and Rural Extension, Rodovia BR-101 Norte, Km. 151, Bebedouro, Linhares 29915-140, ES, Brazil

**Keywords:** NFT, nutrient accumulation, root development, gas exchange

## Abstract

Lettuce (*Lactuca sativa* L.) is a widely consumed leafy vegetable cultivated globally, particularly under soilless systems to enable intensive and high-efficiency production. In these systems, precise nutrient solution management is essential, especially regarding calcium, an indispensable macronutrient involved in cell wall stabilization, membrane integrity, root development, and physiological responses to stress. This study evaluated the morphological and nutritional performance of crisp lettuce cultivars Mônica SF 31 and Veneranda grown using a nutrient film technique (NFT) system, using Calcário, Lithocal^®^, and LT Supra^®^ as alternative calcium sources, alongside a control treatment. The experiment followed a randomized block design in a 4 × 2 factorial arrangement. Measured variables included root, leaf, and stem dry mass; stem length and diameter; and nutrient accumulation and gas exchange parameters. Data were subjected to analysis of variance and means were compared using Scott-knott test. Calcium supplementation significantly increased plant growth, with leaf fresh mass rising by 37% and root fresh mass by 56% compared to the control. Foliar calcium and magnesium concentrations were effectively enhanced by the treatments. Calcário, Lithocal^®^, and LT Supra^®^ demonstrated effectiveness as calcium sources in soilless lettuce cultivation, promoting improved nutrient accumulation—particularly calcium and magnesium—and substantial gains in fresh biomass and root system development. Calcium supplementation is therefore recommended as a strategy to enhance productivity and crop quality in intensive soilless systems.

## 1. Introduction

Lettuce (*Lactuca sativa* L.) is one of the main leafy vegetables cultivated and consumed in Brazil and worldwide, with crisp cultivars being particularly appreciated by populations [1,2]. This species is consumed globally due to its flavor and beneficial properties, serving as a source of bioactive compounds such as chlorophylls, carotenoids, and polyphenols, which can provide health benefits [3,4].

However, lettuce is sensitive to climatic variations, which can compromise plant growth, yield stability, and product quality [1,2]. In this context, the use of soilless culture systems, combined with protected cultivation, enables intensive production with nutritional solutions tailored to each crop, with or without the use of natural and artificial substrates [5].

Nutrient solutions for lettuce in soilless culture can be acquired commercially as standardized, ready-to-use solutions, or they can be prepared by the grower. For formulations prepared by growers, there is a wide variety of substances available that allow the preparation of nutrient solutions capable of meeting the crop’s nutritional demands [6].

In this context, the incorporation of nutritional technologies containing calcium in their composition, such as Calcário, Lithocal^®^, and LT Supra^®^, may represent important alternatives for the nutritional management of lettuce. In addition to acting as mineral sources, some of these products—particularly those derived from biogenic calcareous matrices such as *Lithothamnium* sp.—have been associated with biostimulant effects, capable of modulating physiological processes related to plant growth and nutrient use efficiency. Calcium is an essential macronutrient for plant development and is highly demanded by leafy vegetables, playing crucial roles in cell division, cell wall formation, regulation of the absorption of other nutrients, and cellular signaling [7,8].

Limited calcium availability influences the absorption of essential nutrients like nitrogen and phosphorus, compromising plant development [9]. Furthermore, calcium acts as an important secondary messenger in cell signaling in response to abiotic stresses such as drought, salinity, and extreme temperature variations [9]. Elevated intracellular calcium ion levels activate defense mechanisms, including the production of reactive oxygen species (ROS) and the expression of stress-related genes, increasing plant tolerance to adverse conditions [10]. From a structural perspective, calcium contributes to cell wall robustness, membrane integrity, and enzymatic function, in addition to regulating crucial processes like plant growth and development. It also influences gas exchanges, such as photosynthesis and stomatal conductance [11].

Maintaining optimal calcium levels is thus essential to optimize physiological functionality, stress resilience, and agricultural yield [12]. Its importance is highlighted in lettuce production, where calcium deficiency can lead to various problems, such as physiological disorders and poor production quality [13]. Thus, this study aimed to evaluate the morphological and nutritional characteristics of crisp lettuce cultivars Mônica SF 31 and Veneranda produced in a soilless culture system, using three calcium carbonate-based products: Calcário (Imarcal Indústria de Mármores e Calcário Ltd.a., Vargem Alta, Brazil), Lithocal^®^ (Litho Plant Indústria e Comércio de Fertilizantes Ltd.a., Linhares, ES, Brazil) and LT Supra^®^ (Supramar Minerais Ltda., Serra, ES, Brazil).

Soilless vegetable production systems, including hydroponics, have expanded rapidly as a strategy to intensify yield and standardize quality under protected cultivation. This intensification, however, increases the demand for inputs capable of improving resource-use efficiency and enhancing plant physiological resilience [14]. Within this framework, plant biostimulants have gained prominence due to their capacity to modulate physiological processes associated with nutrient use efficiency, tolerance to abiotic stresses, and product quality [15]. This conceptual approach aligns with the definition established by Regulation (EU) 2019/1009, which characterizes plant biostimulants according to their ability to stimulate plant nutritional processes independently of their direct nutrient content [16]. Recent syntheses further position biostimulants as key tools for advancing sustainable and efficient horticultural systems [17].

Among the factors limiting lettuce performance and marketable quality in intensive systems, calcium (Ca) plays a central role due to its structural function in cell walls, membrane stabilization, and involvement in cellular signaling. Functional Ca deficiencies may occur even when nutrient solution concentrations are adequate, as Ca transport occurs predominantly via the xylem and is closely associated with transpirational water flow [18]. Its limited phloem mobility restricts redistribution to low-transpiring tissues, particularly young and inner leaves [19]. In lettuce, this physiological constraint frequently results in Ca-related disorders affecting expanding tissues with reduced transpiration rates [20].

In this context, products derived from biogenic calcareous matrices, particularly those based on the calcified alga *Lithothamnium* sp., have been proposed as agricultural inputs with potential functional effects beyond simple mineral supply [21,22]. Their relevance is especially pertinent when evaluated from a physiological and nutritional perspective. Recent horticultural reviews emphasize the importance of assessing non-microbial, naturally derived biostimulants as tools to modulate growth, gas exchange, water-use efficiency, and plant nutritional status under controlled environments [17]. Nevertheless, experimental evidence under hydroponic cultivation directly comparing distinct carbonate sources—mineral and algal—and their integrated effects on growth, biomass allocation, physiology, and mineral nutrition remains limited.

Therefore, the present study was conducted under greenhouse conditions using an NFT system with two crisp lettuce cultivars (‘Mônica SF 31’ and ‘Veneranda’) to evaluate whether supplementation of a standard nutrient solution with three calcium carbonate sources—a commercial limestone (Calcário Imarcal) and two products predominantly derived from *Lithothamnium* sp. (Lithocal^®^ and LT Supra^®^)—modifies plant performance at multiple scales. Growth parameters, biomass partitioning, architectural traits, macro- and micronutrient concentrations, and gas exchange variables were integrated to examine the role of calcareous sources, with particular emphasis on *Lithothamnium*-based products, within the broader framework of advances in biostimulant use in horticultural crops.

## 2. Results

### 2.1. Development

**Figure 1:**
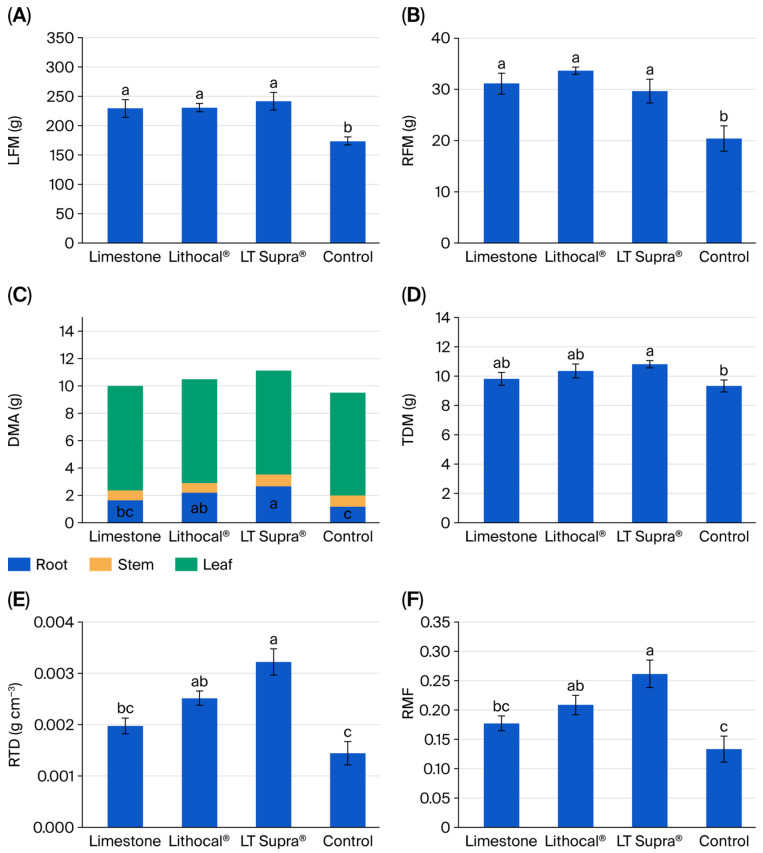
Growth and biomass allocation of lettuce plants cultivated in a nutrient film technique (NFT) system under different calcium carbonate sources (control, limestone, Lithocal^®^, and LT Supra^®^). (**A**) LFM = leaf fresh mass; (**B**) RFM = root fresh mass; (**C**) DMA = dry mass accumulation; (**D**) TDM = total dry mass; (**E**) RTD = root tissue density; (**F**) RMF = root mass fraction. As no significant interaction between calcium sources and cultivars was observed, data are presented as the mean of both cultivars for each calcium treatment. Error bars represent the standard error of the mean (n = 5 blocks). Means followed by the same letter do not differ according to the Scott-Knott test at the 5% probability level.

The development of lettuce plants was influenced by both the cultivars and the products applied in the soilless culture solution; however, no interaction between these factors was observed for any of the analyzed variables. The products similarly affected plant development in both cultivars, influencing leaf and root-related variables (Figure 1). No significant differences were observed for NF, LDM, RI, SSL, and TDM in response to either the products or the cultivars.

### 2.2. Gas Exchanges

**Figure 2:**
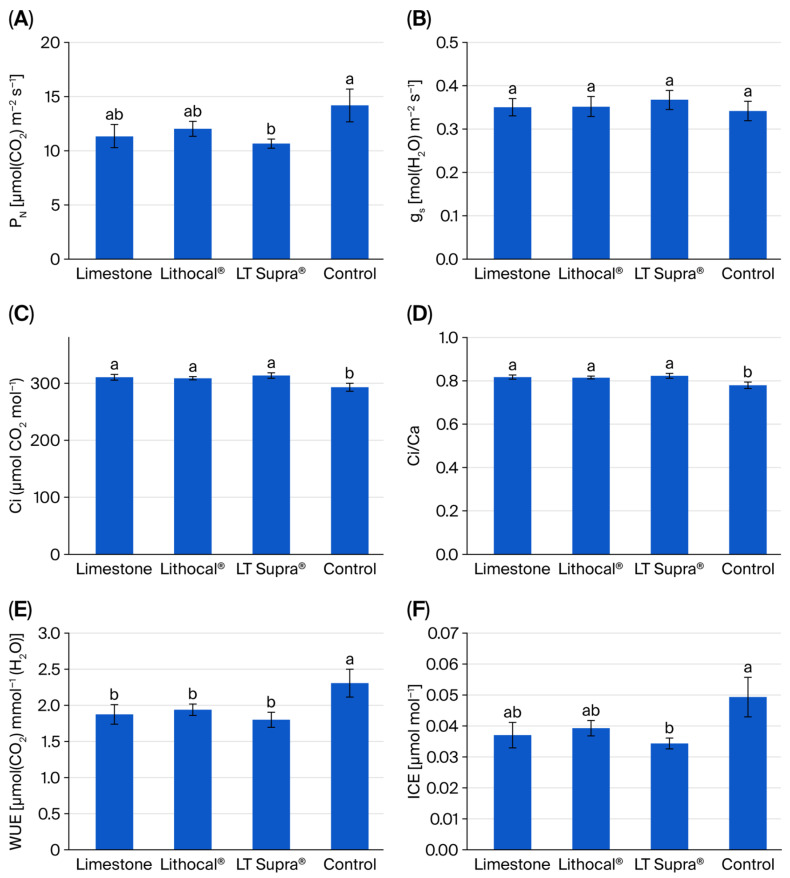
Gas exchange parameters of lettuce plants cultivated in a nutrient film technique (NFT) system under different calcium carbonate sources (control, limestone, Lithocal^®^, and LT Supra^®^). (**A**) PN = net CO_2_ assimilation rate; (**B**) gs = stomatal conductance; (**C**) Ci = intercellular CO_2_ concentration; (**D**) Ci/Ca = ratio between intercellular and ambient CO_2_ concentration; (**E**) WUE = water use efficiency; (**F**) ICE = intrinsic carboxylation efficiency. As no significant interaction between calcium sources and cultivars was observed, data are presented as the mean of both cultivars for each calcium treatment. Gas exchange measurements were performed on two plants per plot, and values represent the mean per experimental unit. Error bars indicate the standard error of the mean (n = 5 blocks). Means followed by the same letter do not differ according to the Scott-Knott test at the 5% probability level.

Gas exchanges were influenced by both the cultivars and the products used in the soilless culture solution; however, no interaction between these factors was observed for any of the analyzed variables. The products affected gas exchanges similarly in both cultivars, influencing the variables PN, Ci, Ci/Ca, WUE, and ICE. Differences between cultivars were detected only for WUE. No significant differences were observed for gs and E. The supplementation with calcium carbonate reduced PN, ICE, and WUE compared to the control but increased Ci and Ci/Ca, with no differences observed among the calcium carbonate sources tested (Figure 2).

### 2.3. Nutrients

**Figure 3:**
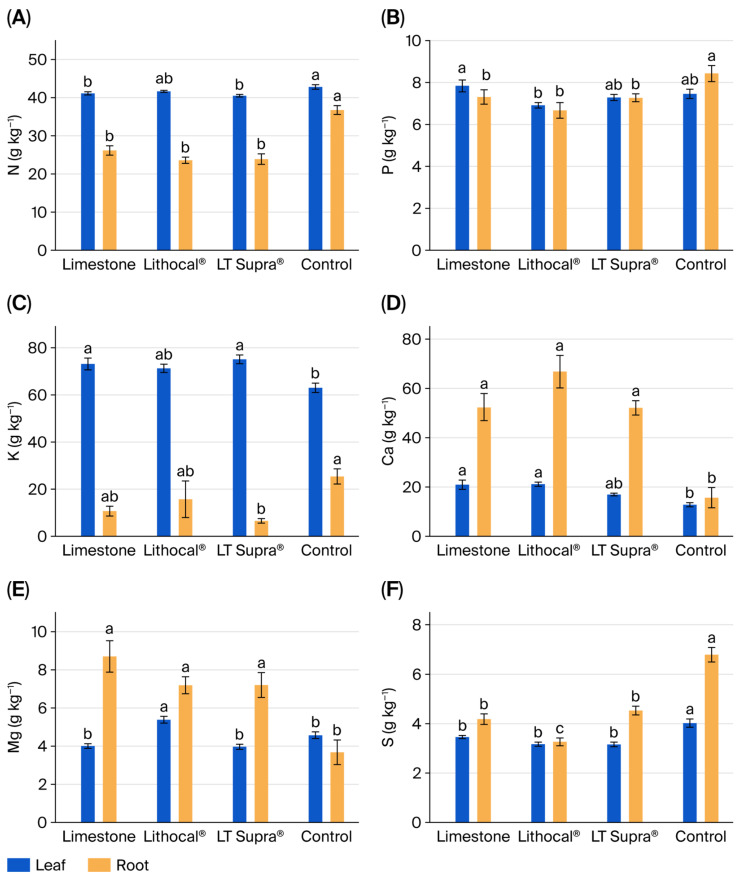
Macronutrient contents in lettuce plants cultivated in a nutrient film technique (NFT) system under different calcium carbonate sources (control, limestone, Lithocal^®^, and LT Supra^®^), for the cultivars Mônica SF 31 and Veneranda. (**A**) Nitrogen accumulation; (**B**) Phosphorus accumulation; (**C**) Potassium accumulation; (**D**) Calcium accumulation; (**E**) Magnesium accumulation; (**F**) Sulfur accumulation. For variables in which a significant interaction between calcium sources and cultivars was observed, results are presented separately for each cultivar. Error bars represent the standard error of the mean (n = 5 blocks). Means followed by the same letter do not differ according to the Scott-Knott test at the 5% probability level.

A significant interaction between products and cultivars was observed only for leaf calcium content and root phosphorus and sulfur content (Figure 3). Calcium carbonate supplementation influenced the contents of all macronutrients in leaves and roots, but affected only foliar copper and root zinc, manganese, and boron. The cultivars affected the contents of potassium, calcium, magnesium, sulfur, and boron in the leaves, and nitrogen, phosphorus, and manganese in the roots. No differences were observed for leaf iron, zinc, and manganese contents, or for root iron and copper contents.

## 3. Discussion

The application of alternative calcium sources significantly enhanced lettuce growth under soilless culture conditions, as demonstrated by increased fresh biomass accumulation in both shoots and roots, as well as higher total dry mass, root tissue density and root mass fraction compared to the control. These responses indicate that calcium supplementation promoted plant vigor primarily through structural and developmental mechanisms, rather than through direct stimulation of photosynthetic activity.

Calcium plays a fundamental role in cell wall stabilization, membrane integrity and regulation of cell expansion and division, processes that directly support tissue growth and biomass accumulation in short-cycle leafy vegetables [7]. Under NFT conditions, where nutrient availability is continuous and root–solution interactions are immediate, these calcium-mediated structural functions become especially relevant for sustaining rapid growth [7,14,23,24].

The significant increases in root fresh mass, root tissue density and root mass fraction observed under calcium-enriched treatments indicate that calcium availability directly influenced root system development. Calcium is known to regulate meristematic activity, influence cell elongation and contribute to the formation of mechanically stable root tissues, resulting in denser and more functionally efficient root systems [7,25,26]. From a nutritional perspective, increased root biomass and density expand the effective absorptive surface area, enhancing nutrient and water uptake and supporting greater shoot development, as classically described in plant mineral nutrition theory [27].

Among the evaluated calcium sources, LT Supra^®^ and Lithocal^®^ promoted greater root dry mass accumulation compared to Calcário and the control. This response may be partially associated with the presence of humic-like substances derived from calcareous algae, which have been reported to exert auxin-like effects on root growth. Humic substances can stimulate cell division, lateral root formation and root elongation through mechanisms involving proton pump activation and modulation of hormonal signaling pathways [28]. Previous studies with *Lithothamnium*-based products have documented enhanced root development and metabolic activity in different crops [21,22]. Although the present study did not directly assess hormonal or molecular pathways, the observed root responses are consistent with these previously described physiological effects, supporting the interpretation of a complementary biostimulant action.

In contrast, plants grown under the control treatment exhibited lower investment in root biomass and tissue density, which may limit nutrient acquisition capacity despite potentially reducing respiratory costs. In high-demand, short-cycle crops such as lettuce, reduced root development can constrain whole-plant growth and biomass accumulation, reinforcing the importance of adequate calcium supply for balanced growth and efficient biomass partitioning [27].

Although calcium supplementation enhanced biomass accumulation, plants grown under calcium-enriched nutrient solutions exhibited lower net photosynthetic rate, intrinsic carboxylation efficiency and water use efficiency, along with increased intercellular CO_2_ concentration and Ci/Ca ratio. This apparent decoupling between photosynthetic parameters and biomass production indicates that growth stimulation was not driven by increased instantaneous carbon assimilation, but rather by improvements in growth efficiency and carbon allocation.

The increase in intercellular CO_2_ concentration in the absence of changes in stomatal conductance or transpiration suggests a biochemical limitation to CO_2_ fixation rather than stomatal restriction. Elevated calcium levels can influence chloroplast ionic balance and enzyme activity, potentially affecting Rubisco efficiency under specific nutritional conditions [11]. However, in leafy vegetables cultivated under soilless systems, biomass accumulation is frequently more dependent on cell expansion, tissue hydration and structural reinforcement than on sustained increases in photosynthetic rate per unit leaf area [23].

Moreover, adequate calcium nutrition improves membrane stability and reduces cellular leakage and metabolic repair demands, allowing a greater proportion of assimilated carbon to be allocated to structural growth rather than maintenance respiration [7]. Similar dissociations between photosynthetic performance and biomass accumulation have been reported in NFT-grown lettuce, where optimized nutrient uptake and root development compensate for moderate reductions in instantaneous gas exchange parameters [29]. Therefore, the observed reductions in photosynthetic indices should be interpreted as physiological adjustments rather than indicators of impaired plant performance.

Calcium supplementation promoted consistent changes in macro- and micronutrient accumulation patterns in both leaves and roots, highlighting the central role of this nutrient in regulating ionic balance within the nutrient solution and plant tissues [14]. In soilless cultivation systems such as NFT, where nutrient uptake occurs under highly dynamic conditions with continuous supply, calcium acts not only as a structural component but also as a modulator of ionic interactions, influencing the availability, absorption, and redistribution of other essential nutrients [30]. In this context, in addition to the expected increase in Ca content, the different calcium sources resulted in variations in the accumulation of potassium, magnesium, nitrogen, phosphorus, and sulfur, indicating integrated nutritional responses rather than isolated effects associated with a single element [28,29].

The higher potassium accumulation observed in calcium-enriched treatments may be related to changes in nutrient solution pH and, consequently, to modulation of ionic availability, as well as to cation competition processes in NFT systems [23,29]. In hydroponic environments, relatively small variations in pH and ionic strength are sufficient to modify the electrochemical gradients involved in root uptake, favoring K^+^ transport under certain conditions [25]. In contrast, the lack of proportional increases in magnesium accumulation is consistent with the well-documented antagonistic interaction between Ca^2+^ and Mg^2+^ during root uptake, as these cations compete for similar transport sites [31]. This behavior reinforces the need to interpret the effects of calcium supplementation from the perspective of overall nutritional balance rather than solely on the basis of its direct effects.

Despite the interactions observed among cations, nitrogen and phosphorus concentrations remained within ranges considered adequate across all treatments, suggesting efficient regulation of uptake and internal allocation of these nutrients under continuous supply conditions. In NFT systems, the maintenance of stable N and P levels indicates that plant metabolism is capable of buffering variations in the ionic balance of the nutrient solution, thereby preserving physiological functioning even when adjustments in mineral composition occur [29].

Taken together, the results indicate that calcium source selection affects not only the accumulation of this nutrient but also the ionic balance of the nutrient solution, root system architecture, biomass partitioning, and nutrient-use efficiency. Although the evaluated calcium sources contributed small amounts of additional nutrients, all treatments were based on the same standard nutrient solution. Furthermore, nutrient solution pH and electrical conductivity were continuously monitored and corrected whenever necessary, maintaining values within the recommended ranges for lettuce cultivation. These management practices reduced potential variations in nutrient availability caused by differences among calcium sources and helped ensure comparable growing conditions throughout the experiment. The superior performance of algae-derived calcium sources suggests their potential as multifunctional inputs, capable of combining mineral nutrition with biostimulant effects previously reported in the literature, thereby contributing to improved growth efficiency and greater physiological stability in soilless cultivation systems.

## 4. Materials and Methods

### 4.1. Experimental Location and Plant Material

The study was conducted in a greenhouse at the Experimental Farm of the Federal University of Espírito Santo, located in the municipality of São Mateus, Northern Espírito Santo State, Brazil, from October to November 2022. The region’s geographical coordinates are 18°40′32″ S latitude and 39°51′39″ W longitude, with an altitude of 37.7 m above sea level. According to Köppen’s classification, the climate is Tropical Aw, characterized by a dry winter. Average temperatures range from 25 to 30 °C during summer and from 19 to 21 °C in winter [32].

The experiment utilized a soilless culture system where lettuce seedlings were cultivated in phenolic foam plates, with each cell measuring 1.9 × 1.9 × 2.0 cm. To eliminate chemical residues from manufacturing, the plates underwent a washing process with deionized water. Three seeds were initially added to each phenolic foam cell. After 5 days, thinning was performed, leaving only the most vigorous seedling, which was then transferred to a germination nursery. The plantlets were irrigated for 16 days with nutrient solution number 02 [33], at ½ ionic strength.

Twenty-one days after sowing, the seedlings were transferred to definitive cultivation benches, employing the Nutrient Film Technique (NFT) system, with a spacing of 25 × 25 cm. They received the nutrient solution with the experimental treatments for an additional 25 days, totaling a 46-day cycle. In this method, water containing essential nutrients for plant development was supplied intermittently to the roots. The NFT system consists of a nutrient solution reservoir, a pumping system, cultivation channels, and a mechanism for returning the solution to the reservoir.

**Figure 4:**
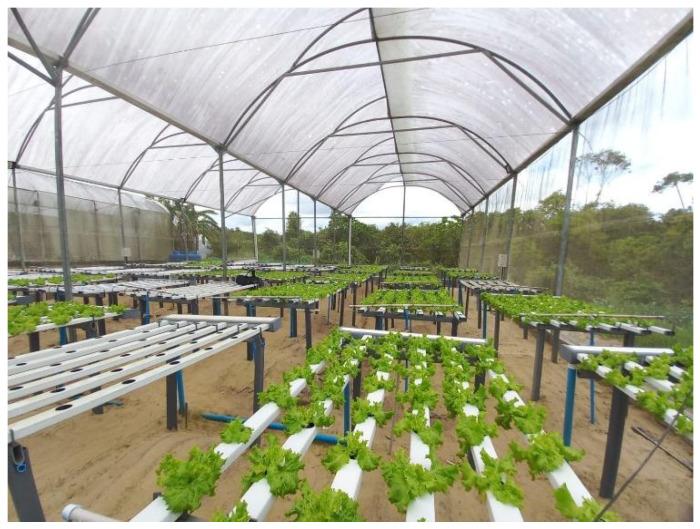
Greenhouse experimental setup used for lettuce cultivation in a nutrient film technique (NFT) soilless system. Lettuce plants (*Lactuca sativa* L.) were grown in hydroponic channels arranged on elevated benches under protected environmental conditions, allowing controlled evaluation of nutrient solutions and calcium sources.

**Figure 5:**
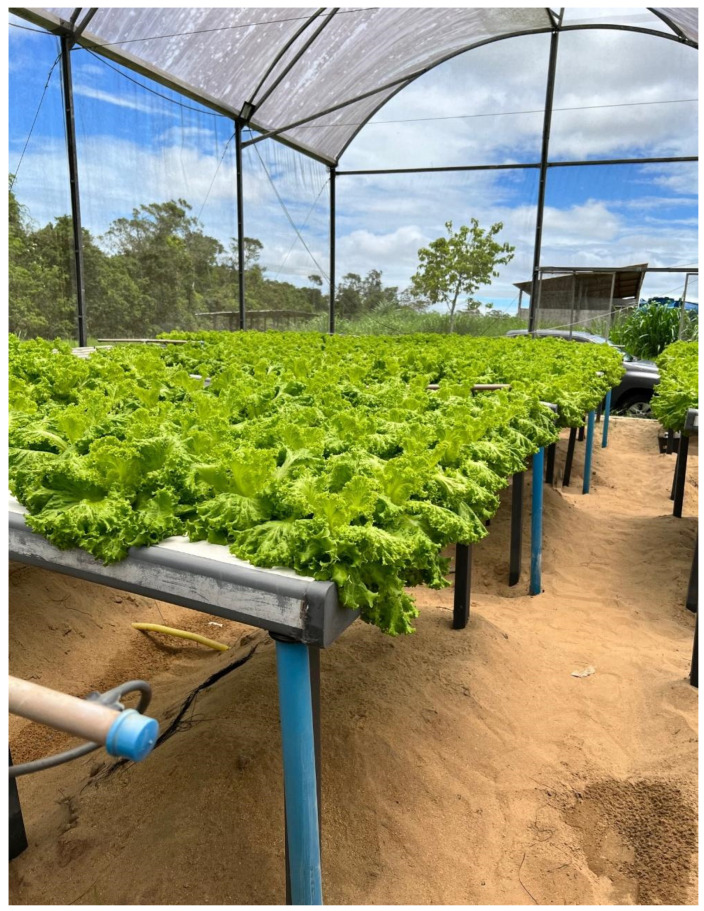
Experimental setup of lettuce cultivation under greenhouse conditions using a nutrient film technique (NFT) system.

The overall greenhouse layout and the NFT cultivation system used in this study are presented in Figure 4 and Figure 5.

### 4.2. Experimental Design and Treatments

Two crisp lettuce cultivars, Mônica SF 31 and Veneranda, were evaluated. These cultivars were selected for their tolerance to high temperatures and resistance to premature bolting, characteristics important for cultivation in warm climates. The choice of these cultivars aimed to assess the performance of varieties adapted to these conditions. Furthermore, Veneranda is distinguished by the light green coloration of its leaves, while Mônica exhibits a medium green hue, allowing for the analysis of the impact of pigmentation variation on plant response to thermal stress.

The standard nutrient solution used in all treatments was solution number 02 proposed by Martinez and Silva Filho [33]. The calcium sources evaluated (Calcário Imarcal, Lithocal^®^ and LT Supra^®^) were added to the same base nutrient solution, and the applied doses were calculated to provide comparable calcium concentrations among treatments. Although these products also contained additional nutrients, particularly magnesium and, in the case of LT Supra^®^, small amounts of nitrogen, phosphorus, potassium and sulfur, the base nutrient solution composition remained identical for all treatments. Throughout the cultivation period, pH and electrical conductivity (EC) were monitored and adjusted to maintain the nutrient solution within the recommended ranges. The pH was measured using a portable pH meter (Incoterm^®^, Porto Alegre, RS, Brazil), with a measurement range of pH 0–14 and a resolution of 0.01 pH units. Electrical conductivity was measured using a portable conductivity meter (model XXX DiST4, Hanna, Limena, Italy).

The experiment was conducted in a randomized complete block design in a 4 × 2 factorial arrangement, consisting of four calcium treatments (control, Calcário Imarcal, Lithocal^®^, and LT Supra^®^) and two lettuce cultivars (Mônica SF 31 and Veneranda). The calcium sources evaluated were: (i) Calcário Imarcal (commercial limestone); (ii) Lithocal^®^, a product derived from the calcareous alga *Lithothamnium* sp., described as a gel-type suspension composed of water, calcium carbonate, and magnesium oxide, containing 18.5% total Ca and 6.0% total Mg [25]; and (iii) LT Supra^®^, obtained from the grinding of rhodoliths predominantly composed of *Lithothamnium* sp., with physicochemical characteristics including 0.06% N, 0.09% P, 0.06% K, 31.19% Ca, 2.06% Mg, and 0.29% S, in addition to micronutrients such as 48.06 mg kg^−1^ B, 0.97 mg kg^−1^ Cu, 14,765.56 mg kg^−1^ Fe, 481.89 mg kg^−1^ Mn, 10.50 mg kg^−1^ Zn, and 8084.48 mg kg^−1^ Na, as well as 9.31% fulvic acid and 0.93% humic acid [21]. The control treatment consisted of the standard nutrient solution without the addition of calcium carbonate sources.

Five blocks were used, with each experimental plot composed of 25 plants, resulting in 125 plants per treatment. The experimental unit corresponded to each plot (block × treatment combination). Each experimental plot consisted of 25 plants; how-ever, five central plants were used for evaluations, and their mean value was considered a single observation for statistical analysis.

The pH of the solution was monitored using a portable pH meter and adjusted to a range of 5.5 to 6.5 using 2N hydrochloric acid (HCl). The electrical conductivity (EC) of the solution was monitored with a portable digital conductometer and adjusted to 2.0 dS m^−1^. Nutrient replenishment was performed based on this measurement, allowing for up to 20% depletion of the initial solution conductivity. Plant water consumption was monitored, permitting a maximum reduction of 20% of the initial volume.

### 4.3. Evaluation of Growth and Development

For development evaluation, five central plants from each plot were collected, ex-cluding border plants. The plants were separated into stems, leaves, and roots. Shoot development was assessed by counting the number of leaves (NF), fresh leaf mass (LFM), dry leaf mass (LDM), leaf mass fraction (LMF), leaf mass per leaf area (LMA), specific leaf area (SLA), stem length (SL), stem diameter (LD), fresh stem mass (SMF), dry stem mass (SDM), stem mass fraction (SMF), specific stem length (SSL), and robustness index (RI). Root development was measured by root tissue density (RTD), dry root system mass (DRM), fresh root system mass (RFM), root mass fraction (RMF), and root volume (RV).

Dry mass allocation, expressed in g, was obtained by weighing the fractionated organs with an analytical precision balance after drying in a forced-air oven at 70 °C until constant weight was achieved. The LMF was obtained by the ratio between LDM and total dry mass (MST). The CC was measured from the collar to the apical bud using a graduated ruler, with results expressed in cm. The DC was determined at the collar region using a digital precision caliper, expressed in mm. The robustness index was calculated as the ratio between CC/DC, with the result expressed in cm cm^−1^.

Specific stem length (SSL) was obtained by dividing stem length by dry stem mass, with the result expressed in mg^−1^ [26]. Stem mass fraction (SMF) was obtained by di-viding dry stem mass by total plant dry mass, expressed in g g^−1^ [34]. Root volume (RV) was obtained by water displacement in a graduated cylinder.

After growth and development evaluations, the dried leaf, stem, and root samples were milled to assess macronutrient and micronutrient [35]. Nitrogen was determined by the sulfuric digestion method with titrimetric determination, while phosphorus and sulfur were determined by nitro-perchloric digestion with spectrophotometric deter-mination. Potassium and sodium underwent nitro-perchloric digestion with flame photometric determination. The relative amounts from the extracts were determined for phosphorus by colorimetry; for potassium by flame photometry; and for sulfur by turbidimetry. Calcium, Magnesium, Iron, Zinc, Copper, and Manganese were deter-mined by nitro-perchloric digestion with atomic absorption spectrophotometry; Boron by incineration (dry method) with colorimetric determination [36].

Gas exchange was evaluated on two plants per plot, selected from the central area of each experimental unit to avoid border effects. Measurements were performed on fully expanded leaves, located in the middle third of the plant canopy, always on the same side of the leaf blade to ensure consistency among samples.

Gas exchange parameters were measured in the morning (07:00–10:00 h) using an infrared gas analyzer (IRGA, LI-6400, LI-COR Inc., Lincoln, NE, USA). Before recording, leaves were allowed to stabilize inside the chamber for an acclimation period of approximately 2–3 min. Measurements were conducted under ambient greenhouse conditions, with natural light as the radiation source. Environmental conditions during measurements were monitored, with average air temperature ranging from 25 to 30 °C and relative humidity between 60 and 80%.

The parameters evaluated included net photosynthetic rate (PN, µmol CO_2_ m^−2^ s^−1^), stomatal conductance (gs, mol H_2_O m^−2^ s^−1^), transpiration rate (E, mmol H_2_O m^−2^ s^−1^), and intercellular CO_2_ concentration (Ci, µmol mol^−1^). Water use efficiency (WUE) and intrinsic carboxylation efficiency (ICE) were calculated from these variables. For each leaf, multiple readings were taken and averaged to obtain a representative value. For statistical purposes, the mean value of the two plants per plot was considered a single biological replicate (n = 5). Data were subjected to analysis of variance (ANO-VA), and when significant, means were compared using the Scott-Knott test at the 5% probability level.

### 4.4. Statistical Analysis

Data were analyzed using the Sisvar statistical software version 6.6 [37]. The assumptions of normality and homogeneity of variances were verified using the Shapiro–Wilk and Levene tests, respectively. When these assumptions were met, the data were subjected to analysis of variance (ANOVA) considering a randomized complete block design in a 4 × 2 factorial arrangement (four calcium sources and two cultivars). The effects of calcium sources, cultivars, and their interaction were evaluated using the F test at the 5% probability level. When significant effects were detected, means were compared using the Scott-Knott test (*p* ≤ 0.05).

## 5. Conclusions

The supplementation of nutrient solutions with calcium carbonate sources improved lettuce performance in the NFT system, promoting greater biomass accumulation, enhanced root development, and increased calcium and magnesium uptake. Products derived from *Lithothamnium* sp. (Lithocal^®^ and LT Supra^®^) were associated with greater root growth and nutrient accumulation compared to limestone, indicating improved growth efficiency under the evaluated conditions. Although these responses are consistent with previously reported effects of algal-derived inputs, the experimental design does not allow the distinction between nutritional and potential biostimulant effects. Therefore, the observed responses should be interpreted primarily as improvements in mineral nutrition, with possible complementary functional effects. Overall, calcareous algal products represent promising calcium sources for soilless cultivation systems, contributing to improved plant performance and nutrient-use efficiency. Further studies are required to isolate and confirm potential biostimulant mechanisms under controlled conditions.

## Data Availability

The original contributions presented in the study are included in the article; further inquiries can be directed to the corresponding authors.

## References

[1] Guimarães C.M., Cunha F.F., Silva F.C.S., Araújo E.D., Guimarães A.B.F., Mantovani E.C., Silva D.J.H. (2019). Agronomic performance of lettuce cultivars submitted to different irrigation depths. PLoS ONE.

[2] Sala F.C., Costa C.P. (2012). Retrospective and trends of lettuce crop in Brazil. Hortic. Bras..

[3] Shi M., Gu J., Wu H., Rauf A., Emran T.B., Khan Z., Mitra S., Aljohani A.S.M., Alhumaydi F.A., Al-Awthan Y.S. (2022). Phytochemicals, nutrition, metabolism, bioavailability, and health benefits in lettuce: A comprehensive review. Antioxidants.

[4] Kottwittz D., Silva V.N. (2024). Cultivo de alface crespa com diferentes fontes de nitrogênio. Ciênc. Agríc..

[5] Almeida T.B.F., Prado R.M., Correia M.A.R., Puga A.P., Barbosa J.C. (2011). Avaliação nutricional da alface cultivada em soluções nutritivas suprimidas de macronutrientes. Biotemas.

[6] Velazquez-González R.S., Garcia-Garcia A.L., Ventura-Zapata E., Barceinas-Sanchez J.D.O., Sosa-Savedra J.C. (2022). A review on hydroponics and the technologies associated for medium- and small-scale operations. Agriculture.

[7] White P.J., Broadley M.R. (2003). Calcium in plants. Ann. Bot..

[8] Hepler P.K., Winship L.J. (2010). Calcium at the cell wall–cytoplast interface. J. Integr. Plant Biol..

[9] Kang X., Zhao L., Liu X. (2024). Calcium signaling and the response to heat shock in crop plants. Int. J. Mol. Sci..

[10] Campos F.G., Seixas D.P., Barzotto G.R., Jorge L.G., Ducatti K.R., Ferreira G., Rodrigues T.M., Silva E.A.A., Boaro C.S.F. (2022). Roles of calcium signaling in gene expression and photosynthetic acclimatization of *Solanum lycopersicum* Micro-Tom after mechanical damage. Int. J. Mol. Sci..

[11] Hu L., Zhang J., Gao Y., Ma N., Chang Y., Jie J. (2023). Effects of calcium on photosynthetic characteristics, yield and quality of soilless culture celery. North. Hortic..

[12] Gupta S., Kaur N., Kant K., Jindal P., Ali A., Naeem M. (2023). Calcium: A master regulator of stress tolerance in plants. S. Afr. J. Bot..

[13] Yamamoto E.L.M., Silva F.A., Costa M.R., Oliveira R.M. (2011). Função do cálcio na degradação da parede celular vegetal de frutos. Rev. Verde Agroecol. Desenvolv. Sustentável.

[14] Savvas D., Gruga N. (2018). Application of soilless culture technologies in the modern greenhouse industry—A review. Eur. J. Hortic. Sci..

[15] Du Jardin P. (2015). Plant biostimulants: Definition, concept, main categories and regulation. Sci. Hortic..

[16] European Parliament (2019). Regulation (EU) 2019/1009 of the European Parliament and of the Council of 5 June 2019 laying down rules on the making available on the market of EU fertilising products and amending Regulations (EC) No 1069/2009 and (EC) No 1107/2009 and repealing Regulation (EC) No 2003/2003. Off. J. Eur. Union.

[17] Rouphael Y., Colla G. (2020). Toward a sustainable agriculture through plant biostimulants: From experimental data to practical applications. Agronomy.

[18] Wdowiak A., Podgórska A., Szal B. (2024). Calcium in plants: An important element of cell physiology and structure, signaling, and stress responses. Acta Physiol. Plant..

[19] Moosavi-Nezhad M., Gholami M., Rouphael Y., Colla G. (2025). A calcium-mobilizing biostimulant provides tipburn control in lettuce by enhancing calcium transport and leaf distribution. Front. Plant Sci..

[20] Kang S., Kim H., Park S. (2016). Effects of light intensity and growth rate on tipburn development and leaf calcium concentration in butterhead lettuce. Sci. Hortic..

[21] Ramos E.P., Ferreira T.R., Aguiar D.B., Alves F.L., Dousseau Arantes S. (2023). *Lithothamnion* sp. as biostimulant in plant cultivation. Pesqui. Agropecu. Trop..

[22] Amatussi J.O., Mógor Á.F., Cordêiro E.C.N., Mógor G., Marques H.M.C., Lara G.B. (2022). Synergic combination of calcareous algae and cyanobacteria stimulate metabolic alterations improving plant growth and yield. Res. Sq..

[23] Samarakoon U.C., Palmer J., Ling P., Altland J. (2020). Effects of electrical conductivity, pH and foliar application of calcium chloride on yield and tipburn of *Lactuca sativa* grown using the nutrient-film technique. HortScience.

[24] Sonneveld C., Voogt W. (2009). Plant Nutrition of Greenhouse Crops.

[25] Marschner P. (2012). Marschner’s Mineral Nutrition of Higher Plants.

[26] Weng X., Li H., Ren C., Zhou Y., Zhu W., Zhang S., Liu L. (2022). Calcium regulates growth and nutrient absorption in poplar seedlings. Front. Plant Sci..

[27] Lynch J.P., Bassirirad H. (2005). Root architecture and nutrient acquisition. Nutrient Acquisition by Plants: An Ecological Perspective.

[28] Cannelas L.P., Olivares F.L. (2014). Physiological responses to humic substances as plant growth promoter. Chem. Biol. Technol. Agric..

[29] Vought K., Bayabil H.K., Pompeo J., Crawford D., Zhang Y., Correll M., Martin-Ryals A. (2024). Dynamics of micro and macronutrients in a hydroponic nutrient film technique system under lettuce cultivation. Heliyon.

[30] Thor K. (2019). Calcium—Nutrient and messenger. Front. Plant Sci..

[31] Li H., Liu F., Zhang X., Gao J., Chen P. (2024). Magnesium deficiency or excess hinders tomato growth, potassium and calcium uptake. Plant Soil Environ..

[32] Alvares C.A., Stape J.L., Sentelhas P.C., Gonçalves J.L.M., Sparovek G. (2014). Köppen’s climate classification map for Brazil. Meteorol. Z..

[33] Martinez H.E.P., Silva Filho J.B. (2006). Introdução ao Cultivo Hidropônico de Plantas.

[34] Poorter H., Niklas K.J., Reich P.B., Oleksyn J., Poot P., Mommer L. (2011). Biomass allocation to leaves, stems and roots: Meta-analyses of interspecific variation and environmental control. New Phytol..

[35] EMBRAPA (2009). Sistema de Produção de Alface.

[36] Malavolta E., Vitti G.C., Oliveira S.A. (1997). Avaliação do Estado Nutricional das Plantas: Princípios e Aplicações.

[37] Ferreira D.F. (2011). Sisvar: A computer statistical analysis system. Ciênc. Agrotecnol..

